# Supplementary material for: ACE2 binding is an ancestral and evolvable trait of sarbecoviruses
Source: Nature. 2022 Feb 3;603(7903):913–8. doi: 10.1038/s41586-022-04464-z (PMC8967715; doi:10.1038/s41586-022-04464-z)

nature portfolio
https://doi.org/10.1038/s41586-022-04464-z

Supplementary information

ACE2 binding is an ancestral and evolvable trait of sarbecoviruses

In the format provided by the
authors and unedited

Supplementary Figure 1

Uncropped images from Extended Data Figure 3c.

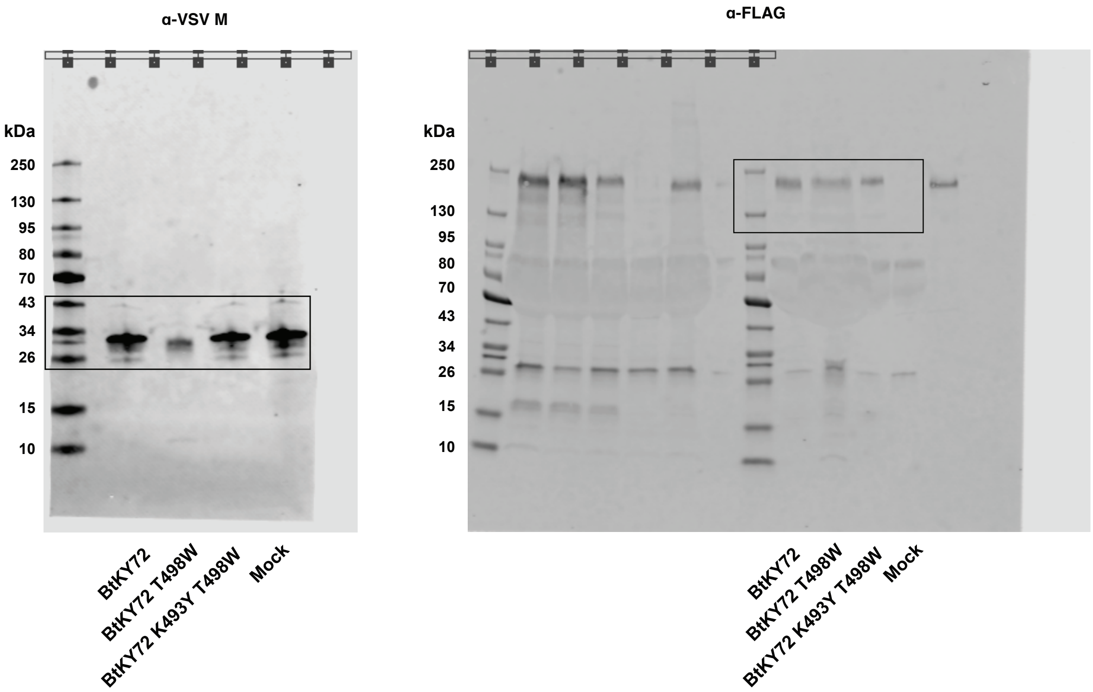

Supplement: Supplementary file 1 — Uncropped images of the western blots in Extended Data Fig. 3c. [file 41586_2022_4464_MOESM1_ESM.pdf]
