# Supplementary material for: ACE2 binding is an ancestral and evolvable trait of sarbecoviruses
Source: Nature. 2022 Feb 3;603(7903):913–8. doi: 10.1038/s41586-022-04464-z (PMC8967715; doi:10.1038/s41586-022-04464-z)
Supplement: Supplementary file 2 — Reporting Summary [file 41586_2022_4464_MOESM2_ESM.pdf]

nature portfolio

Corresponding author(s): Starr, Veesler, Bloom

Last updated by author(s): Dec 12, 2021

Reporting Summary

Nature Portfolio wishes to improve the reproducibility of the work that we publish. This form provides structure for consistency and transparency in reporting. For further information on Nature Portfolio policies, see our Editorial Policies and the Editorial Policy Checklist.

Statistics

For all statistical analyses, confirm that the following items are present in the figure legend, table legend, main text, or Methods section.

n/a	Confirmed	
☐	☒	The exact sample size (*n*) for each experimental group/condition, given as a discrete number and unit of measurement
☐	☒	A statement on whether measurements were taken from distinct samples or whether the same sample was measured repeatedly
☒	☐	The statistical test(s) used AND whether they are one- or two-sided<br>*Only common tests should be described solely by name; describe more complex techniques in the Methods section.*
☒	☐	A description of all covariates tested
☒	☐	A description of any assumptions or corrections, such as tests of normality and adjustment for multiple comparisons
☐	☒	A full description of the statistical parameters including central tendency (e.g. means) or other basic estimates (e.g. regression coefficient) AND variation (e.g. standard deviation) or associated estimates of uncertainty (e.g. confidence intervals)
☒	☐	For null hypothesis testing, the test statistic (e.g. *F*, *t*, *r*) with confidence intervals, effect sizes, degrees of freedom and *P* value noted<br>*Give P values as exact values whenever suitable.*
☒	☐	For Bayesian analysis, information on the choice of priors and Markov chain Monte Carlo settings
☒	☐	For hierarchical and complex designs, identification of the appropriate level for tests and full reporting of outcomes
☒	☐	Estimates of effect sizes (e.g. Cohen's *d*, Pearson's *r*), indicating how they were calculated

Our web collection on statistics for biologists contains articles on many of the points above.

Software and code

Policy information about availability of computer code

Data collection	* Cell sorting experiments were operated using BD FACSDiva software (v. 8.0.2), and flow cytometry data processed in FlowJo (v. 10)
Data analysis	* phylogenetics and bioinformatics software includes mafft (v. 7.471), PAL2NAL (v. 14), RAxML (v. 8.2.12), FastML (v. 3.11) and GARD (v. 0.2)<br>* PacBio sequences were analyzed with ccs (v. 5.0.0) and alignparse (v. 0.1.6)<br>* Illumina sequences were processed with dms_variants (v. 0.8.5), and analyzed with fitdistrplus (v. 1.0.14)<br>* All custom code used for data analysis is available on GitHub: https://github.com/jbloomlab/SARSr-CoV_homolog_survey<br>* A summary of the computational pipeline and links to individual notebooks detailing steps of analysis is available on Github: https://github.com/jbloomlab/SARSr-CoV_homolog_survey/blob/master/results/summary/summary.md. Specific notebooks are listed below:<br>* All steps of bioinformatic analysis, including specific programmatic commands, alignments, raw data, and output files can be found on GitHub: https://github.com/jbloomlab/SARSr-CoV_homolog_survey/tree/master/RBD_ASR<br>* The PacBio CCS processing pipeline is available on GitHub: https://github.com/jbloomlab/SARSr-CoV_homolog_survey/blob/master/results/summary/process_ccs.md<br>* The full pipeline for computing per-barcode DMS expression values is described on GitHub: https://github.com/jbloomlab/SARSr-CoV_homolog_survey/blob/master/results/summary/compute_expression_meanF.md<br>* The full pipeline for computing per-barcode DMS binding affinities is described on GitHub: https://github.com/jbloomlab/SARSr-CoV_homolog_survey/blob/master/results/summary/compute_binding_Kd.md<br>* The full pipeline for barcode collapsing to final variant/mutant scores is described on GitHub: https://github.com/jbloomlab/SARSr-CoV_homolog_survey/blob/master/results/summary/barcode_to_genotype_phenotypes.md.

For manuscripts utilizing custom algorithms or software that are central to the research but not yet described in published literature, software must be made available to editors and reviewers. We strongly encourage code deposition in a community repository (e.g. GitHub). See the Nature Portfolio guidelines for submitting code & software for further information.

Data

Policy information about availability of data

All manuscripts must include a data availability statement. This statement should provide the following information, where applicable:

- Accession codes, unique identifiers, or web links for publicly available datasets
- A description of any restrictions on data availability
- For clinical datasets or third party data, please ensure that the statement adheres to our policy

* PacBio circular consensus sequences are available from the NCBI SRA, BioSample SAMN18316101
* Illumina sequences for barcode counting are available from the NCBI SRA, BioSample SAMN20174027
* Table of measurements of ACE2 binding and expression for all parental RBDs is available on GitHub: https://github.com/jbloomlab/SARSr-CoV_homolog_survey/blob/master/results/final_variant_scores/wt_variant_scores.csv
* Table of measurements of ACE2 binding and expression for all single mutant RBDs is available on GitHub: https://github.com/jbloomlab/SARSr-CoV_homolog_survey/blob/master/results/final_variant_scores/mut_variant_scores.csv
* All virus names, species and location of sampling, and sequence accessions (GenBank, GISAID) or citations are provided on GitHub: https://github.com/jbloomlab/SARSr-CoV_homolog_survey/blob/master/RBD_ASR/RBD_accessions.csv.

Field-specific reporting

Please select the one below that is the best fit for your research. If you are not sure, read the appropriate sections before making your selection.

☒ Life sciences ☐ Behavioural & social sciences ☐ Ecological, evolutionary & environmental sciences

For a reference copy of the document with all sections, see nature.com/documents/nr-reporting-summary-flat.pdf

Life sciences study design

All studies must disclose on these points even when the disclosure is negative.

Sample size	No sample size determination was performed, as we were not performing statistical tests dependent on appropriate sample size determination
Data exclusions	No data were excluded from analyses
Replication	High-throughput titration measurements were replicated with two independently constructed gene libraries (Extended Data Fig. 2g). BLI binding assays were replicated in three batches of purified protein. Pseudovirus entry assays were replicated with two or three independent batches of pseudovirus generation. All experimental points are shown for DMS assays and pseudoviral entry assays, showing replication of results. Representative BLI traces are shown but were replicated, including when replicating under different sample concentrations (Extended Data Fig. 3a,b).
Randomization	Randomization was not performed. We conducted a standard survey of measurements across a panel of genotypes, which is not a study design that requires randomization
Blinding	Blinding was not performed in our study. High throughput titration experiments are conducted in massively parallel bulk experiments where there is no identifiability of individual variant genotypes, so blinding is not a relevant experimental attribute.

Reporting for specific materials, systems and methods

We require information from authors about some types of materials, experimental systems and methods used in many studies. Here, indicate whether each material, system or method listed is relevant to your study. If you are not sure if a list item applies to your research, read the appropriate section before selecting a response.

Materials & experimental systems

n/a	Involved in the study	
☐	☒	Antibodies
☐	☒	Eukaryotic cell lines
☒	☐	Palaeontology and archaeology
☒	☐	Animals and other organisms
☒	☐	Human research participants
☒	☐	Clinical data
☒	☐	Dual use research of concern

Methods

n/a	Involved in the study	
☒	☐	ChIP-seq
☐	☒	Flow cytometry
☒	☐	MRI-based neuroimaging

Antibodies

Antibodies used	FITC-conjutaged chicken anti-c-Myc (Immunology Consultants Lab, CYMC-45F); PE-conjugated streptavidin (ThermoFisher S866); iFluor-647-conjugated mouse anti-His (Genscript A01802); PE-conjugated goat anti-human IgG (Jackson ImmunoResearch Labs 109-115-098); mouse anti-VSV G (ATCC CRL-2700); Alexa Fluor 680-conjugated AffiniPure goat anti-mouse IgG (Jackson ImmunoResearch 115-625-174); mouse monoclonal anti-FLAG M2 antibody (Sigma F3165); Anti-VSV-M [23H12] antibody (Kerafast EB0011)
Validation	No validation was performed

Eukaryotic cell lines

Policy information about cell lines

Cell line source(s)	* Expi293F: ThermoFisher A14527<br>* HEK293T: ATCC CRL-11268<br>* HEK293T-ACE2: Crawford, KHD et al. Protocol and reagents for pseudotyping lentiviral particles with SARS-CoV-2 spike protein for neutralization asasys. Viruses 12 (2020).
Authentication	Cell lines were not authenticated
Mycoplasma contamination	Cell lines were not tested for mycoplasma contamination
Commonly misidentified lines<br>(See ICLAC register)	No commonly misidentified lines were used.

Flow Cytometry

Plots

Confirm that:

☒ The axis labels state the marker and fluorochrome used (e.g. CD4-FITC).

☒ The axis scales are clearly visible. Include numbers along axes only for bottom left plot of group (a 'group' is an analysis of identical markers).

☒ All plots are contour plots with outliers or pseudocolor plots.

☒ A numerical value for number of cells or percentage (with statistics) is provided.

Methodology

Sample preparation	Yeast libraries expressing a library of sarbecovirus RBD variants on the cell surface were induced using standard culture techniques, as described in the Methods
Instrument	Sorting was conducted on a BD FACSAria II cell sorter. Flow cytometry analysis was conducted on a BD LSRFortessa X50 flow cytometer.
Software	Cell sorting experiments were operated using BD FACSDiva software (v. 8.0.2), and flow cytometry data processed in FlowJo (v. 10)
Cell population abundance	We were not sorting a specific target population, but rather partitioning all cells into encompassing bins on the basis of expression or ACE2 labeling, for downstream sequencing and reconstruction of per-variant labeling.
Gating strategy	Single cells were selected via FSC/SSC, FSC-W/FSC-A, and SSC-W/SSC-A gating. RBD-expressing cells were gated using a FITC/FSC gate. Single, RBD+ cells were sorted into bins of fluorescence on the basis of unlabeled or labeled control cells expressing the unmutated SARS-CoV-2 RBD. Representative gating schemes are illustrated in Extended Data Fig. 2b-d.

☒ Tick this box to confirm that a figure exemplifying the gating strategy is provided in the Supplementary Information.
